# The economic burden of lung cancer in low- and lower-middle-income countries: a systematic review

**DOI:** 10.1186/s13690-025-01738-6

**Published:** 2025-10-13

**Authors:** Elyesa Ünal, Elena Goodchild, Volker Winkler, Stephan Brenner, Andreas Deckert, Peter Dambach, Olaf Horstick, Andrea Kaifie, Valérie R. Louis

**Affiliations:** 1 Institute for Occupational, Social, and Environmental Medicine, Medical Faculty, RWTH Aachen University, Aachen, Germany; 2 Heidelberg Institute of Global Health, University Hospital and Medical Faculty, University of Heidelberg, Heidelberg, Germany

**Keywords:** Lung cancer, Lung neoplasms, Economic burden, Direct costs, Indirect costs, Low-income countries, Lower-middle-income countries, Developing countries

## Abstract

**Background:**

Lung Cancer is one of the four most frequent malignant neoplasms in the world, and first in mortality. In 2020, lung cancer resulted in roughly 1.8 million deaths globally. Lung cancer is a major public health problem in low and lower-middle income countries, where high rates of tobacco smoking, environmental pollution, and limited access to healthcare resources contribute to a significant burden of the disease. The objective of our review was to compare costs among available countries and identify major cost drivers.

**Methods:**

A systematic database search was performed according to PRISMA guidelines on Web of Science, PubMed, Cochrane, EBSCO, ISPOR, and Google Scholar using a pre-defined search string. Studies were screened for eligibility and included if they provided information about the direct and indirect costs of lung cancer in low- and lower-middle-income countries. Search cut off was on August 4, 2025. The data from included studies were extracted, quantitatively synthesized, and compared.

**Results:**

Of 2,383 articles, 15 met the inclusion criteria. All studies were conducted in low- and lower-middle-income countries, with a time frame ranging from 2003 to 2025. Most studies estimated costs based on prevalence. Most studies reported total healthcare costs as direct costs related to lung cancer; seven studies reported on indirect costs. Total average direct costs ranged from USD 2,540.66 per patient in Nepal to USD 10,179.98 per patient in the first year of treatment in Bolivia. Indirect costs varied from USD 140,995,744.13 in Bangladesh to USD 320,427,043.97 in Vietnam for their given respective sample sizes. Cost estimates varied substantially even for the same country depending on study’s analytical focus, timeframe, and other methodological considerations.

**Conclusion:**

In conclusion, the costs of lung cancer are substantial and increase with disease progression, encompassing both direct and indirect components. High out-of-pocket expenditures further intensify the economic burden on patients and may lead to an underestimation of the true societal costs. The lack of standardized methodologies and robust data highlights the need for further research to guide effective policy and resource allocation in LMICs.

**Trial registration:**

Registration: PROSPERO, ID CRD42020160370.

**Supplementary Information:**

The online version contains supplementary material available at 10.1186/s13690-025-01738-6.

**Table:** 

**Text box 1. Contributions to the literature**
• This review is the first to synthesize available evidence on the economic burden of lung cancer in low- and lower-middle-income countries.
• The findings reveal major gaps in cost reporting, comparability, and methodological transparency, which hinder evidence-based health policy.
• The results highlight the urgent need for standardized cost reporting frameworks to guide resource allocation and strengthen cancer control strategies in resource-limited settings.

## Introduction

Lung cancer is the leading cause of cancer incidence and mortality worldwide. In 2020, there were around 2.2 million new cases of lung cancer and 1.8 million deaths, making it the most lethal cancer worldwide [1]. Although the incidence of cancer is generally higher in high-income countries, the mortality rates are higher in low-income countries, where access to early detection and treatment is often limited. In 2012, an estimated 65% of all cancer deaths occurred in low- and middle-income countries (LMICs), and this burden is expected to rise to 75% by 2030 [2].

The future burden of lung cancer in men (in low- and medium-Human Development Index countries) and women (globally) will depend largely on the smoking habits of the population, including the duration of smoking, the extent of smoking prevention, the type of tobacco smoked, and consumption patterns [3]. The leading risk factor for lung cancer mortality is smoking, which accounted for 64.2% of all lung cancer deaths worldwide in 2019 [4].

Globally, smoking caused 7.69 million deaths and 200 million disability-adjusted life-years in 2019 [5]. According to estimates, almost one billion people worldwide smoke tobacco and 336 million use smokeless tobacco, most of whom live in low- to middle-income countries [6]. In these countries, the average number of pack-years per smoker is 17.1, smoking prevalence is higher in rural than in urban areas, and rates are generally greater among individuals with the lowest levels of education and household wealth. Consequently, smoking cessation rates are also lower among the poorest and socially disadvantaged groups [6]. Other major risk factors for lung cancer mortality include air pollution, which contributes to 15.1% of lung cancer deaths worldwide, occupational exposure to carcinogens (such as asbestos and diesel exhaust), and household air pollution from solid fuels, which particularly affects women in low-income regions. Additional risk factors are exposure to passive smoking, residential radon, high fasting plasma glucose (suggesting a metabolic link), and a diet low in fruit [4].

Initially, patients often show no symptoms, especially in the case of peripheral carcinomas. Cough, dyspnea, hemoptysis, and chest pain, weight loss occurs as the condition progresses [7]. More than half of patients are diagnosed at an advanced stage, precluding curative treatment [8, 9]. A recent systematic review found no clear or negative association between delays in diagnosis/treatment and survival, and no association between longer delays and earlier stage diagnosis. High-income countries (e.g., UK, US, Denmark) have implemented fast-track programs, multidisciplinary teams, and other interventions to reduce the time to treatment, but their effectiveness varies [10]. In LMICs, lung cancer is often diagnosed late due to limited access to healthcare, low awareness of the disease, weak medical infrastructure, and inefficient referral systems [11, 12]. One review categorized the factors affecting delays in lung cancer diagnosis in LMICs as patient delay, physician delay and health system delay. Patient delay was ignoring symptoms by patients; physician delay was mainly misdiagnosis of lung cancer as tuberculosis and misinterpretation of chest CT by physicians, but also lower suspicion of lung cancer. Health system delay was the time spent waiting at each interval due to the inability of the health system to accommodate all patients. The high cost and inaccessibility of diagnostic tests such as CT scans, bronchoscopy, chemotherapy, and chest radiography may contribute to their underuse and, in turn, to delays in starting treatment even after a diagnosis has been made [13]. The challenges in the diagnosis of the condition under consideration are multifaceted and include a significantly high rate of false positives resulting from the presence of various granulomatous diseases that present with similar initial symptoms as lung cancer [14]. Additionally, the limited availability of CT scans and PET CT facilities pose significant obstacles, primarily attributable to economic constraints and feasibility issues related to large-scale implementation [14]. Therefore, the disease is typically diagnosed at an advanced stage with poor outcomes resulting from compromised treatment effectiveness. Treatment approaches to small-cell carcinoma depend on the respective cancer stage. Early disease stages I and II are usually treated with surgery and a combination of chemotherapy. Once in a metastasizing stage, combined chemotherapy and prophylactic cranial irradiation are the preferred methods of treatment [7, 14]. Early stage non-small-cell lung cancers are also surgically removed and subsequently treated with adjuvant chemotherapy [15]. Treatment strategies for non-small cell lung cancer have evolved from cytotoxic chemotherapy to targeted therapies and immunotherapies [16].

As lung cancer is most often diagnosed at an advanced stage, this can have a significant impact on the overall cost of the disease to society. In Europe, the cost of lung cancer is higher than that of breast, colorectal or prostate cancer and accounts for 15% of the total economic cost of cancer [17]. A large body of literature has quantified the economic burden of lung cancer in high- and high-middle-income countries [18–21]. A prominent example is a recent systematic review of the economic burden of small-cell lung cancer (SCLC) on healthcare costs across Australia, Canada, Italy, France, Turkey, United Kingdom, Japan, and the United States [19]. Several direct cost components have been identified as potential cost drivers for the economic burden of SCLC: chemotherapy, diagnostic costs, treatment costs, medication costs, or surgical costs (hospitalization, nursing care, emergency admissions, follow-up appointments, and inpatient and outpatient care). The authors of the publications included in the review selected the direct cost components based on their specific objectives, making direct comparisons impossible. In the US, costs related to chemotherapy are USD 896.73 per session or USD 10,760.85 for a course of treatment [19]. The average cost of treatment in Australia varies between USD 12,688 and USD 19,046, depending on the stage of SCLC and the mortality rate [19, 22]. The average medical cost per patient in Turkey was USD 5,480 ± 4,088. The study also estimated the indirect costs. Indirect costs, such as loss of productivity (e.g., absenteeism), care costs (indirect costs associated with care and housework), out-of-pocket expenses, and loss of income due to early retirement due to illness, have been reported to range USD 500 to USD 99,000 per patient in Turkey [19, 23]. Another review reported the burden of disease by reporting costs of illness from several European countries, the United Kingdom, the United States, Canada, but also from some high-middle-income countries such as Korea, China, and Iran [24]. However, there is no systematic review focusing only on LMICs. To close this research gap, this study aimed to systematically review the costs of lung cancer in low- and lower-middle-income countries. Specifically, the goal was to evaluate and assess the direct and indirect costs of lung cancer, as well as to assess key drivers on healthcare costs.

## Methods

### Approach

**Fig. 1:**
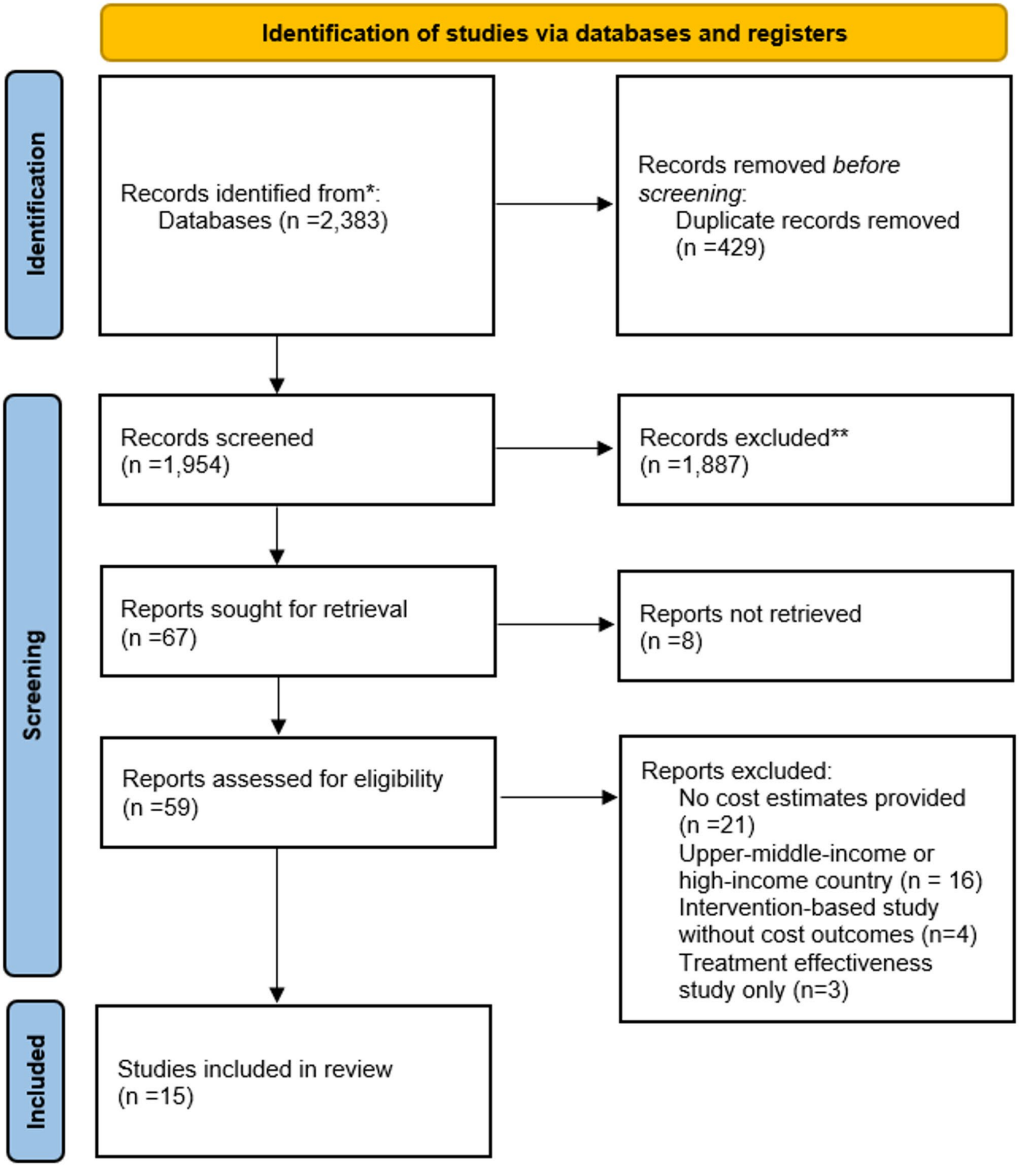
PRISMA flow chart of study selection

The review adopted the Preferred Reporting Items for Systematic Review and Meta-Analysis (PRISMA) statement. A pre-defined protocol was submitted to the International Prospective Register of Systematic Reviews (PROSPERO) under the identification number: CRD42020160370 [25, 26]. A systematic literature search was conducted using six databases: PubMed, Web of Science, EBSCO, International Society for Pharmacoeconomics and Outcomes Research (ISPOR), Cochrane, and Google Scholar (only the first 200 hits). The last search was completed on August 4, 2025. A flow chart illustrating our selection process is shown in Fig. 1.

### Search terms

The search utilized a combination of terms and their synonyms in three major categories: economic burden, lung cancer, and low- and lower-middle-income countries (LIC & LMIC). When searching each category, we included its medical subject heading term (MeSH) and its possible spelling alterations (supplementary Table 1).

### Eligibility criteria

The definition of eligibility criteria was based on PRISMA recommendations [25] and included only studies reporting on individuals with lung cancer conducted in low- and lower-middle-income countries. Studies had to report information on direct or indirect costs. Only peer-reviewed studies were considered. Abstracts or presentations that had been published without a full-text article were excluded. The systematic literature review encompassed research spanning from 1960 to 2025. No language restrictions were imposed, but the search was performed in English and required articles being indexed in English. The World Bank definition was used to include countries with low- and lower middle-income economies as those with a gross national income (GNI) per capita in 2024 of USD 1,135 or less and between USD 1,136 and USD 4,495, respectively [27].

### Screening procedure

Three stages of review were conducted for the purpose of inclusion: title assessment, abstract assessment, and full-text assessment (Fig. 1). After removal of duplicates, all identified studies were screened by EÜ and EG independently on the basis of titles and abstracts using the online screening tool Rayyan [28]. Then, full texts of articles that matched the eligibility criteria were further screened. Reference lists from included studies were also screened for potential additional relevant studies. Any discrepancies were resolved by discussion among all authors.

### Data extraction

A pre-designed data extraction form was developed based on the review objectives and the PRISMA guidelines. The form was piloted on a subset of studies and adjusted for clarity and comprehensiveness. Key study characteristics were captured, including author name, publication year, country, population data, study design, cost types (direct/indirect), data sources, methods of cost estimation and timeframe of cost reporting (supplementary Table 2). Data extraction was performed by a single reviewer and cross-checked for accuracy and completeness. Reported costs were grouped into direct (medical and non-medical) and indirect costs (supplementary Table 3). We report the costs from the societal perspective, which includes all aspects that are important to society and its functioning [29]. From the societal perspective, cost components are considered regardless of who bears them and who is affected by the consequences of an intervention [30]. Direct medical costs were defined as expenses related to the prevention, diagnosis, treatment, and follow-up of lung cancer, including consultation, diagnostic imaging, laboratory tests, hospital care, surgery, chemotherapy, radiotherapy, targeted therapy, immunotherapy, and palliative therapy. Direct non-medical costs included patient-incurred expenses such as transportation, accommodation, and food during health care visits. Indirect costs referred to productivity losses due to morbidity and premature mortality, including income lost by family members who provide informal patient care, and were calculated using the Human Capital Approach or other reported methods [29].

### Conversion of economic data

To enable cross-country comparisons and adjust for variations in inflation and purchasing power, all cost data were converted to 2022 United States dollars (USD) using the CCEMG–EPPI-Centre Cost Converter [31]. For studies reporting costs in the national currency, the reported amount and cost year were entered directly into the tool. If costs were reported in USD, they were first converted into the relevant local currency using the exchange rate mentioned in the study and then processed in the converter. Where the reference year for the cost was not reported, the year prior to data collection was assumed as the price year. The tool applies a two-stage adjustment [32, 33]. Stage one adjusts for inflation using the Gross Domestic Product (GDP) deflator, which captures general price changes across all economic sectors within a country. Stage two then converts the inflation-adjusted value into the target currency using purchasing power parity (PPP) exchange rates, ensuring that differences in price levels between countries are appropriately accounted for. This method enables meaningful and comparable assessments of the economic burden of lung cancer across diverse health systems and economies.

### Quality assessment

All included studies were assessed for their scientific quality as recommended by the Equator Network using the Consolidated Health Economic Evaluation Reporting Standards (CHEERS) statement published in 2013 [34, 35]. In addition to this quality assessment tool, a point system for categorizing the results that has been applied in other economic studies was used [36–38]. One point was awarded once criteria were fully met, half a point for partially met criteria, and zero points if no or inadequate information was provided. The label ‘not applicable’ identifies studies in which the checklist did not report the described criterion or recommendation. A percentage rating was determined by summing the points for each study. Studies with a score higher than 75% were categorized as good, 50–74% as moderate, and below 50% as poor quality. The quality assessment was performed by two independent authors in a manner that ensured the absence of any mutual influence or bias.

## Results

### Literature search results

A total of 2,383 eligible publications were initially identified through the database searches (Fig. 1). After removing duplicates, 1,954 potentially relevant publications were screened for title and abstract. Of these, 67 were sought for retrieval. Next, 59 full texts were assessed for eligibility, and 44 were excluded, mainly owing to the absence of cost estimates (*n* = 21), the study being conducted in an upper-middle-income or high-income country (*n* = 16), intervention-based studies without cost outcomes (*n* = 4), or studies focusing solely on treatment effectiveness (*n* = 3).

Fifteen studies published between 2003 and 2025 which met the inclusion criteria were included in the analysis [39–53].

### General methodological and study characteristics

**Fig. 2:**
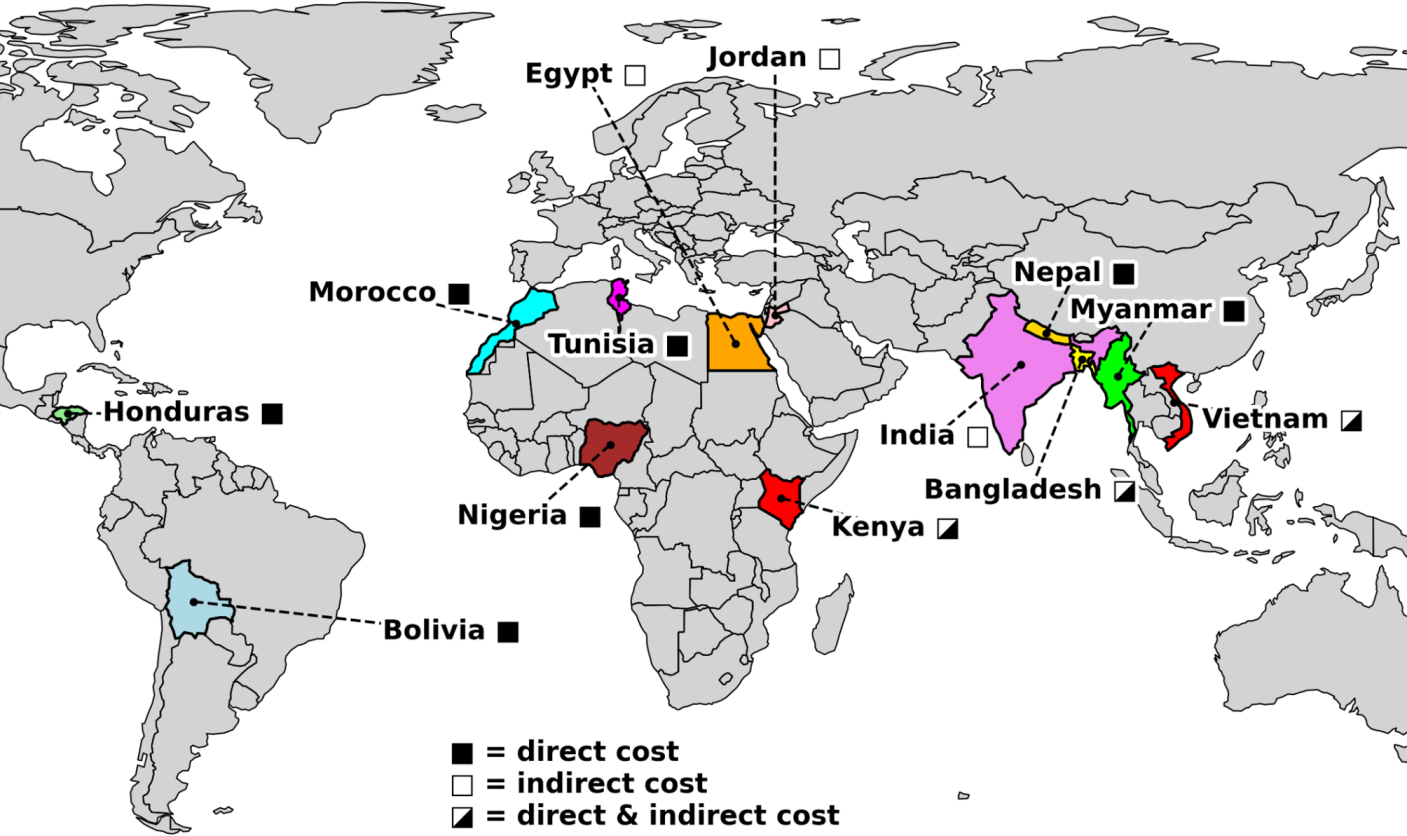
Countries Included in the Review and Type of Economic Burden Assessed

According to the World Bank classification based on Gross National Income (GNI) per capita for 2024 (applicable for fiscal year 2025), all studies included in this review were conducted in lower-middle-income countries (LMICs) (GNI USD 1,136 and USD 4,495 [25]). Thirteen studies reported cost estimates for a single country, covering a broad range of nations including Bangladesh, Egypt, Jordan, Morocco, Kenya, Myanmar, Nepal, Nigeria, Vietnam, and Tunisia. For Vietnam, Morocco, and Tunisia, two studies were available for each country. In addition, two studies covered multiple countries and provided data for LMICs such as Bolivia, Honduras, and India. An overview of the included countries and the cost estimates reported per country is presented in Fig. 2. Data sources varied and included hospital records [39–42, 44, 47], cancer registries [44], literature reviews [45], surveys, and official governmental statistics [48]. Most studies reported estimated average patient costs annually or over the duration of illness [40, 41, 47–49, 51–53], with some providing demographic breakdowns based on sample size, gender, and age [39–42, 44, 45, 48]. Eight studies presented data disaggregated by sex, with one focusing on death as an outcome [39–41, 43, 44, 46, 47]. The average age of samples was reported in six studies [40, 41, 43, 51, 52], while a maximum age limit was set in another three [39, 46, 48]. All papers provided information on the currency used and the publication year, with additional details such as the length of hospital stay mentioned in one paper [52]. Eight studies reported solely on direct costs of lung cancer [39, 43–47, 51, 52], while three focused exclusively on indirect costs [42, 49, 50]. Four studies presented data on both direct and indirect costs [40, 41, 48, 53]. Most studies did not further specify the type of lung cancer (non-small-cell lung cancer or small-cell lung cancer), except for one study published in Tunisia [46].

**Table 1:** Summary of study characteristics

1st author, Publication year [reference]	Country	LIC / LMIC	Setting of study	Sample Size (*n*)	Sample Sex	Samples Age	Study Period	Quality score [40]
Khatiwoda, 2019 [39]	Nepal	LMIC	Bhaktapur Cancer Hospital	57	24 ♂ 33 ♀	≤ 29 - ≥ 60	17/11/2016–13/02/2017	95% Good
Hoang Anh, 2014 [40]	Vietnam	LMIC	6 national hospitals, 5 provincial hospitals, 2 district-level hospitals:	727	538 ♂ 189 ♀	> 18 Ø age 63	03/2011–10/2011	87% Good
Ross, 2007 [41]	Vietnam	LMIC	1 district hospital, 1 provincial hospital, 3 national hospitals	180	130 ♂ 50 ♀	Ø age 58	01/2005–06/2005	68% Moderate
Pearce, 2018 [42]	Brazil, Russia, India, China, South Africa (BRICS)	LMIC	GLOBOCAN data and OECD (Organisation for Economic Co-operation and Development)	25,899 †	18,912 ♂ † 6,987 ♀ †	/	2012	95% Good
Fenniche, 2011 [43]	Tunisia	LMIC	L’hôpital Abderrahmen Mami de l’Ariana	153	/	Ø age 58,7 (PV) Ø age 57,5 (PG)	01/01/2006–30/06/2007	47% Poor
Tachfouti, 2012 [44]	Morocco	LMIC	Grand-Casablanca-region Cancer register; including recovered data from university teaching Hospital Casablanca, private clinics, and private laboratories of pathology	3,336	1,503 ♂ 1,833 ♀	/	2004	63% Moderate
Pichon-Riviere, 2020 [45]	Bolivia, Honduras	LMIC	Review of scientific literature databases	405 Bolivia 366 Honduras	/	/	2015	95% Good
Harizi, 2017 [46]	Tunisia	LMIC	(Hôpital Abderrahmen Mami de l’Ariana)	549	489 ♂ 58 ♀	21–91, Ø age 61,45	06/2008–06/2010	85% Good
Kyaing N, et al., 2003 [47]	Myanmar	LMIC	Medical Records Department of Yangon General Hospital and the radiotherapy unit	3,059	2.130 ♂ 929 ♀	/	1991–1999	75% Good
Faruque, et al., 2020 [48]	Bangladesh	LMIC	Survey; World Health Organization, Country Office for Bangladesh, 2009; Ministry of Health and Family Welfare, Government of Bangladesh, 2017	157,669	/	> 30	01/2018–04/2018	77% Good
Wahab KA, et al., 2025 [49]	Egypt	LMIC	Wage Indicator database World Bank; WHO	5,657	4,013 ♂ † 1,644 ♀ †	/	2019	87% Good
Rashdan O, 2024 [50]	Jordan	LMIC	Global Burden of Disease study 2019; World Bank Group; WHO	general population for respective year	/	/	1990–2019	95% Good
El Harch I, et al., 2023 [51]	Morocco	LMIC	Oncology center	271	253 ♂ 18 ♀	62.5 ± 9.5	2019	80% Good
Olumide AO, 2021 [52]	Nigeria	LMIC	University hospital	5	3 ♂ 2 ♀	59.8 (± 10.6)	09/2016- 01/ 2017	60% Moderate
Mwai D, et al., 2024 [53]	Kenya	LMIC	4 national public hospitals	202	107 ♂ 95 ♀	/	2021–2022	60% Moderate
LIC low-income country; LMIC lower-middle income country; (n) amount; ♂ males; ♀ females; † deaths; ø average; ≤ younger than or equal to; ≥ older than or equal to; p-v therapy with cisplatin + vinerolebine; p-g therapy with cisplatine + gemcitabine; / not reported; GLOBOCAN global cancer observatory; IHME institute for health metrics and evaluation								

### Studies reporting on total healthcare expenditures

The majority of studies utilized total healthcare expenditures per patient as a composite measure of both direct (including medical and non-medical costs) and indirect costs for their respective sample populations, presented in either the local currency or converted to US dollars [39, 40, 44, 47, 48].

Several studies reported health care costs per patient. Many studies focused primarily on estimating direct medical costs per patient, e.g., Tunisia, Morocco, or total direct costs (medical and non-medical) e.g., Nepal for their samples. Some studies, such as those in Vietnam, attempted to quantify both direct and indirect economic burdens. These were often presented as separate components or totals, rather than as a single, consistently defined ‘total health expenditure per patient’ that included all direct and indirect costs. Furthermore, the definition and scope of ‘health expenditure’ varied between studies, with some focusing on costs within a specific time period or setting (e.g., inpatient care).

### Studies reporting on direct cost components

Almost all studies included a wide range of direct expenditure types depending on the authors’ study objectives, such as inpatient and outpatient treatment costs. Cost drivers identified as contributing to the economic burden of lung cancer included several components (Supplementary Table 3). The Tunisian study estimated direct costs and found that chemotherapy was the most important direct cost component (46%), followed by the cost of hospitalization (19%). Other direct cost components mentioned, with their percentage of total direct costs, were imaging (16.4%), surgery (2.4%), radiotherapy (3.8%), other exploratory procedures (2.1%) [46]. The Vietnam study estimated both direct and indirect costs. Direct costs included medical costs (fees, overheads, drugs not included in fees) and non-medical costs (transport, supplementary food) [40]. A study from Nepal aimed to calculate the direct cost of health service utilization for cancer, including medical and non-medical costs comprised costs of consultation, diagnostic investigations, treatment (chemotherapy, radiotherapy, surgery, palliative, and supportive care), and hospital care. The study also reported the mean direct cost by type of treatment, showing variations in medical costs depending on whether the patient received surgery, chemotherapy, radiotherapy, or palliative care [39]. This study from Nigeria reported both direct medical costs, such as hospital stays, clinic visits, examinations, medical procedures, medications and consumables, and non-medical costs, such as transport costs [52]. The Moroccan study estimated the average cost per patient by treatment protocol (first-line chemotherapy, second-line chemotherapy, radiotherapy, surgery, palliative care, other treatments) and stage of diagnosis.

It also reported the total direct medical costs of newly diagnosed lung cancer cases by stage (See Supplementary Table 3 for further details) [44]. A total of eleven studies reported on direct health expenditures as shown in Table 2 [39–41, 43–48]. Only three papers reported costs for males and females separately [42, 45, 49].

One paper reported on the direct costs in the first year and the following year after diagnosis in Bolivia and Honduras [45]. Two papers reported on government settlements, insurance reimbursements, and personal expenses [40, 45]. Another two papers reported on direct out-of-pocket expenses [41, 53].

**Table 2:** Reported direct costs of lung cancer converted to the corresponding 2022 USD value

Country [reference]	LIC / LMIC	Year and currency reported	Time period and sample sizes (*n*)	Total Ø expenditures per patient	Costs for inpatient per patient	Costs for outpatient visit per patient	Reported total expenditures (for given sample size	
Nepal [39]	LIC	2017 Nepalese Rupees / USD 1 USD = 108 NRs	17/11/2016–13/02/2017 *n* = 57	USD 2,540.66	/	/	USD 144,817.43	
Vietnam [40]	LMIC	2011 Vietnamese Dong / USD	03/2011–10/2011 *n* = 47,128	/	USD 3,900.90	USD 412,86	USD 179,517,089.63	
Vietnam [41]	LMIC	2005 Vietnamese Dong 1 USD = 15,000 VN$	01/2005–06/2005 *n* = 11,546	/	USD 1,126.15	/	USD 9,593,933.33	
Tunisia [43]	LMIC	2010 Tunisian Dinar 1 USD = 1.4 TND	06/2008–06/2010 *n* = 549	USD 3,451.64	/	/	USD 1,895,424.81	
Morocco [44]	LMIC	2004 Moroccan dirham/ USD 10 MAD = 1.3 USD	2004 *n* = 3,510	USD 6,483.68 (Stage I, II, IIIA) USD 4,820.47 (Stage IIIb, IV)	/	/	USD 16,919,861.85	
Bolivia [45]	LMIC	2015 Venezuelan Bolívares / USD 1 USD = 6.91 Bs	2015 *n* = 405	USD 10,179.98 (1st year); USD 13,162.01 (2nd year)	/	/	USD 13,114,119.80	
Honduras [45]	LMIC	2015 Honduran Lempira /USD 1 USD = 21.95 HNL	2015 *n* = 366	USD 7,066.80 (1st year); USD 8,143.40 (2nd year)	/	/	USD 7,754,436.06	
Tunisia [46]	LMIC	2007 Tunisian Dinar	01/01/2006–30/06/2007 *n* = 153	USD 3,396.47 (P-G); USD 1,654.43 (P-V)	/	/		
Myanmar [47]	LMIC	1999 Kyats	1991–1999 *n* = 207	/	USD 6,494.56	USD 115.64	USD 125,099.14	
Bangladesh [48]	LMIC	2018 Bangladeshi taka	01/2018–04/2018 *n* = 157,669	/	USD 569.33	USD 819.56	USD 243,569,290.35	
Morocco [51]	LMIC	2019 USD 1 USD = 9,595 MAD	2019 *n* = 271	USD 12,965.64 range (11,749.51- 14,181.83) Stage I 18,402.15 ± 10,050.26 Stage II 20,541.71 ± 7,855.42 Stage III 16,763.44 ± 10,869.48 Stage IV 11,095.89 ± 9,417.01	/	/	/	
Nigeria [52]	LMIC	2020 1USD= ₦ 360.50	09/2016- 01/ 2017 *n* = 5	USD 4,370.86 (USD 4,150.61 direct medical & USD 220.25 direct non-medical cost)	/	/	/	
Kenya [53]	LMIC	2022 USD 115KES = 1US$.	2021–2022 *n* = 202	USD 46,282.23	/	/	/	
LIC low-income-country; LMIC lower-middle-income-country (N) amount of given sample sizes; Ø average; ♂ male; ♀ female; P-V therapy with cisplatin + vinerolebine; P-G therapy with cisplatine + gemcitabine; ₦ naira; KES Kenyan shilling Ø average; / not reported								

### Studies reporting on indirect cost components

Seven of the included studies reported on indirect costs [40–42, 48–50, 53] (Table 3). The indirect costs were generally defined as the monetary value of productivity losses resulting from lung cancer-related morbidity and mortality in the included studies. However, the scope and methodological approaches varied. The two main approaches were: the Human Capital Approach (HCA) [40, 42, 49, 50] and the Value of a Statistical Life Year (VSLY) [53]. The study from Jordan [50] reported the costs using both approaches, while the study from Bangladesh [48] study adopted an expanded human capital approach (HCA) as part of the cost-of-illness (COI) methodology. The HCA method calculates lost productivity by multiplying the years of life lost (YLL), years of productive life lost (YPLL), or lost Disability-Adjusted Life Years (DALYs) by the average wage or Gross National Income per capita (GNIpc), adjusted for discounting (typically 3%). The VSLY method monetises DALYs by assigning a value (often adapted from estimates in high-income countries) to each year of healthy life lost. VSLY is usually derived by dividing VSL by life expectancy and adjusting for income levels using elasticity coefficients.

Four papers reported on both morbidity and mortality costs [40, 48, 50, 53]. Morbidity costs represent productivity losses due to sick leave and disability, while mortality costs include the value of lost lifetime due to premature death from the disease. The study from Vietnam in 2007 [41] reported only on morbidity costs, while the studies from Egypt only reported on mortality costs [49].

**Table 3:** Reported indirect costs of lung cancer converted to the corresponding 2022 USD value

Country [reference]	LIC/LMIC	Year and currency reported	Time period and sample sizes (*n*)	Reported Total Indirect Costs	Morbidity Costs	Mortality Costs
Vietnam [40]	LMIC	2011 Vietnamese Dong / USD	03/2011–10/2011 *n* = 47,128	USD 320,427,043.97*	USD 133,724,399.19*	USD 186,824,433.84*
Vietnam [41]	LMIC	2005 Vietnamese Dong 1 USD = 15,000 VN$	01/2005–06/2005 *n* = 11,546	/	USD 134.22 per admission	/
India [42]	LMIC	2012 USD	2012 *n* = 25,899 †	USD 742,076,370.00 (♂ USD 654,843,560.00; ♀ USD 87,352,307.00) Ø per death USD 28,654.94	/	/
Bangladesh [48]	LMIC	2018 Bangladeshi taka	01/2018–04/2018 *n* = 157,669	USD 140,995,744.13	Annual loss per patient USD 3,653.57 Ø daily loss per patient USD 15,75	/
Egypt [49]	LMIC	2019 USD	2019	/	/	USD 38,561,532.89♂ USD 15,992,843.12 ♀ USD 9,608,80 per death
Jordan [50]	LMIC	2019 international $, purchasing power parity (PPP))	1990–2019 general Population for respective year	USD 41,181,953.30	/	/
Kenya [53]	LMIC	2022 USD	2022 *n* = 202	USD 3,024,239.43 range (2,357,187.91–3,691,290.95)	/	/

LIC low-income country; LMIC lower-middle-income country; * smoking-attributable indirect costs; ♂ males; ♀ females; ø average; / not reported; † death

### Cost estimates of lung cancer

Table 2 shows the direct costs reported in the included studies. The reported total cost estimate varied considerably between countries. The direct inpatient costs per patient vary between USD 569.33 in Bangladesh and USD 3,900.90 in Vietnam to USD 6,494.56 in Myanmar. A study from Nepal revealed total costs of USD 2,540.66 per patient [39]. In Morocco, direct costs for stage I, II, and IIIa were USD 6,483.68 per patient in the first year, and USD 4,820.47 per patient for stage IIIb and IV [44]. A study in Bolivia found that the average expenditure per patient was USD 10,179.98 in the first year, rising to USD 13,162.01 in the second year [45]. Studies from Vietnam, Myanmar, and Bangladesh showed that the costs for inpatient care (USD 3,900.90 / USD 6,494.56 / USD 569.33) differ enormously from those for outpatient care (USD 412.86 / USD 115.64 / USD 819.56) [41, 47, 48]. Furthermore, cost estimates in the same country (Vietnam) varied widely, ranging from USD 3,900.90 to USD 1,126.15 per hospitalization [40, 41].

Indirect cost components were generally less frequently reported compared to direct cost components owing to the varying costing perspectives of the authors. Given the definitions used across studies, indirect costs are usually higher than the direct costs. In Table 3 we further distinguish morbidity and mortality costs. Whereas morbidity costs represent wages lost by individuals who are unable to perform their work due to illness, mortality cost include the value of lifetime lost prematurely from the disease. Total indirect costs ranged from USD 140,995,744.13 in Bangladesh to USD 742,076,370.00 in India for their respective sample sizes [42, 48]. For lung cancer specifically, the Vietnam study provided a breakdown of morbidity costs (USD 133,724,399.19) and mortality costs (USD 186,824,433.84). The BRICS countries (including India and China) focused specifically on estimating productivity losses among people of working age. Liver and lung cancer have the largest impact on total productivity losses in the BRICS countries due to their high incidence. A study from Bangladesh considered the indirect costs of morbidity and mortality, considering the lost time of both employed and non-employed individuals.

### Quality of reporting

Overall, the quality assessment based on the CHEERS checklist revealed that all studies were fully applicable to our research question [34, 35]. Ten studies were assessed to have a reporting quality of “good” based on predetermined criteria [39, 40, 42, 45–51]. Four studies were classified as having “moderate” reporting quality [41, 44, 52, 53], while one study was rated as having “poor” reporting quality [43]. Four studies scored 95% or higher, indicating excellent reporting quality [39, 42, 45, 50] (Supplementary Table 4).

## Discussion

To the best of our knowledge, this is the first systematic analysis to review the economic impact of lung cancer in low- and lower-middle-income countries and to evaluate and assess its direct and indirect costs. All of the studies included in this review were conducted in lower-middle-income countries (LMICs), with the majority reporting direct medical costs. However, only four of the studies explicitly included direct non-medical costs, such as transportation and informal caregiving. None of the studies provided cost estimates disaggregated by non-small-cell and small-cell lung cancer subtypes. Only two studies from Morocco reported costs stratified by cancer stage. The majority of the studies were conducted in African and Asian countries. Despite applying standardised currency and price-year adjustments, cost estimates varied across studies, likely due to differences in the components included in the cost calculations and methodological approaches.

A very comprehensive review of lung cancer costs revealed a wide range of direct costs, from USD 4,484.13 in Korea to USD 45,364.48 in the Netherlands. The examined studies were from high-income countries or upper-middle-income countries (HICs or UMICs) [24]. This is consistent with the results of our own study, which also found a very wide range of direct costs. This review from UMICs and HIC showed that hospital stays accounted for the largest proportion of direct medical costs, at 71% in Switzerland, 65.32% in China, and 64% in Spain. This was followed by chemotherapy and surgery [24]. In contrast, the findings of our review differed significantly. In Tunisia, for example, chemotherapy accounted for 46% of direct medical costs [46]. In Morocco, chemotherapy was also the largest cost driver, accounting for 19.9% [51]. Another Moroccan study reported that radiotherapy represented 68% of total direct medical costs, making it the largest proportion [44]. A study from Nepal found that the cost of treatment (including medicines, therapies, and surgeries) accounted for 72% of direct medical costs [39]. In Kenya, the main cost driver was the cost of medicines [53]. These differences may be partly explained by variations in health system structures and pricing. In high-income countries, hospital stays tend to be significantly more expensive due to higher labour, infrastructure, and service costs. In contrast, medication and treatment-related costs often dominate in LMICs because hospital services are less costly.

### Out of the pocket expenditure

In several of the included LMICs, out-of-pocket (OOP) expenditure places a significant financial burden on patients and households. In Nepal, 86.1% of patients reported experiencing financial hardship due to cancer-related costs. Of these patients, 78.2% took out loans and 47.6% sold property to cover out-of-pocket expenses. The average direct cancer-related costs exceeded patients’ average annual income, indicating financial catastrophe. Even the government subsidy of NPR 100,000 (approximately USD 925.92) was insufficient to prevent financial distress [39]. In Vietnam, for example, a study found that although 80% of operational medical costs (e.g., tests, medicines, and laboratory fees) were covered by insurance, patients still had to pay 20% out of pocket, particularly for non-medical expenses such as transport, food, accommodation, and informal payments [40]. Previous research from Vietnam also indicated that inpatient care costs were primarily paid for directly by patients [41]. In Nigeria and Kenya, OOP payments remain the primary means of financing care for non-communicable diseases, including lung cancer [52, 53]. Such costs have been described as catastrophic, especially given that the minimum monthly income in Nigeria is below USD 85. In Myanmar, patients pay for services such as drugs, diagnostics, and equipment [47]. In Bangladesh, World Bank data show that nearly 14% of the population spends over 10% of their household income on OOP healthcare, pushing 4.5% of them below the poverty line [48]. Evidence from Nigeria suggests that the lack of financial protection, whereby patients bear the full cost of treatment, leads to delayed diagnosis and care-seeking behavior. High out-of-pocket expenses result in financial hardship and may also contribute to poor clinical outcomes by delaying access to timely cancer care. The ACTION (Asean CosTs In Oncology) study group, which assesses the economic impact of cancer reported that over 75% of patients with cancer (with lung cancer being included as a subgroup) in Southeast Asia experienced death within a year of diagnosis or financial catastrophe due to high out-of-pocket expenses [54]. These patterns highlight the urgent need to strengthen protection mechanisms in LMICs to mitigate the economic vulnerability of affected households. This can be achieved by expanding insurance coverage, increasing government funding, and integrating lung cancer care into national health benefit packages.

### Risk factor- tobacco

Tobacco remains the number one risk factor for lung cancer. Due to the global increase in smoking since 1980, the burden of lung cancer is expected to continue rising in the coming years, particularly in developing countries [55]. The vast majority of current smokers – up to 80% – live in low- or middle-income countries, and more than half of all lung cancer deaths occur in less developed regions [55, 56]. This review includes several studies which identify tobacco use (i.e., smoking) as the main risk factor for lung cancer. In Latin America, for example, tobacco use accounted for 79% of lung cancer deaths and 80% of associated medical costs [45]. In Morocco, smoking accounts for 8% of all deaths, 75% of which are due to lung cancer [51]. A study in Kenya showed that tobacco was the main risk factor for lung cancer, accounting for 61% of cases [53]. Given that smoking is a major contributor to lung cancer, effective strategies such as tobacco taxation, prohibiting smoking in public places, limiting tobacco advertising, and providing treatment for tobacco dependence have proven to be beneficial for improving public health. Additionally, smokers with lower incomes tend to be more sensitive to price increases resulting from taxation and have a greater tendency to quit or reduce their smoking habits in response to such measures [57, 58]. The World Health Organization estimates that 48% of men and 10% of women globally are smokers [55]. The studies included in the review consistently confirm that men in the countries studied have a significantly higher smoking prevalence than women. In Tunisia, 50% of men over the age of 25 smoke. Among lung cancer patients, 91.6% were smokers, predominantly male [46]. Nationwide, the smoking prevalence among Moroccans is 13.4%, with 26.9% of men and only 0.4% of women smoking [51]. In Vietnam, 87% of male patients were current or former smokers, compared to 11% of female patients [41]. In Kenya, tobacco consumption is significantly higher among men (19.1%) than women (4.5%) [53]. Although the prevalence of smoking is similar among men in developed and developing countries, it is significantly lower among women in the latter. In areas where smoking rates among women are low, non-tobacco risk factors are likely to play a more significant role in the development of lung cancer (e.g., the inhalation of smoke from charcoal, heating, or cooking) [55]. However, given that smoking is the leading risk factor for lung cancer, and smoking prevalence remains significantly higher among men in many lower-middle-income countries (LMICs), targeted tobacco control efforts — particularly gender-sensitive anti-smoking campaigns — are crucial. Public health strategies should focus on prevention through awareness, education, and regulation, particularly among men.

### Limitations of the study

The heterogeneity in reporting on study design, study objective, methodology varied among studies and made direct comparisons challenging and limited the scope of the study. Lung cancer was often studied as a subgroup of cancer rather than an independent topic, resulting in less detailed information.

Some studies focused only on direct costs, and even these were not always reported as medical and non-medical costs. The studies also used different approaches, with some using retrospective hospital data rather than estimates. And estimates were sometimes based on smoking-attributable fraction (SAF) or population-attributable risk (PAR). Some studies used an SAF to calculate the proportion of indirect costs associated with lung cancer that could be attributed to smoking. This means that the indirect costs associated with lung cancer cases due to causes other than smoking would not be included in their estimates of the indirect costs of lung cancer.

The stage of lung cancer, one of the most important prognostic factors, was not reported in all included studies. This approach would provide a more granular understanding of the economic burden associated with each type and stage of lung cancer, facilitating targeted interventions and resource allocation tailored to the specific need of patients. Instead of categorizing patients by disease stage, they were mainly classified based on treatment pattern, which is a proxy for disease stage. In addition, small-cell lung cancer and non-small-cell lung cancer, which differ in terms of costs and 5-year survival rates, were not fully described, and differentiated in the included studies. This lack of stratification by lung cancer subtype (NSCLC versus SCLC) and by disease stage represents a limitation, as these groups differ markedly in clinical course, treatment costs, and prognosis. Comparisons on a macro level were often challenging due to differences in the medical providers and health care systems. There were a few studies in which the quality of the provided information did not permit converting the cost results to a fixed-price-year, as recommended by the Cochrane Collaboration. There is limited information concerning cancer care and infrastructure in lower-middle-income countries due, in part, to the fact that the health care infrastructure in these countries has traditionally been focused on communicable diseases. Since most studies reported on patients’ progress while they were in treatment, it is possible that the actual cost of treatment might be higher than what has been documented in the studies. Study findings on indirect costs rarely include information about the total number of samples, so comparisons were limited.

### Recommendations and conclusions

This systematic review on the economic burden of lung cancer across lower-middle-income countries showed that the great variety of methodologies and focus on outcomes in the included studies indicate that more research following standardized guidelines is needed. Strengthen national cancer registries and data collection systems in LMICs to obtain more accurate and representative data on lung cancer incidence, prevalence, treatment patterns, and associated costs.

Current estimations of the economic burden of lung cancer in LMICs are likely underestimates due to limitations in data availability and methodological constraints, particularly the frequent exclusion of indirect costs and the challenges in capturing comprehensive direct costs. Further research in low- and middle-income countries (LMICs) is urgently needed to disaggregate lung cancer costs by histological subtype, particularly distinguishing between non-small cell lung cancer (NSCLC) and small cell lung cancer (SCLC). Our review could not include any studies from low-income countries (LICs), which highlights a critical research gap that must be addressed in order to inform equitable health financing and policy planning.

In lower-middle-income countries, lung cancer imposes a significant financial burden on patients and their households, often resulting in excessive out-of-pocket spending and economic hardship. Thus, removing financial barriers, providing financial protection against lung cancer costs, and improving access to care are crucial. Cancer screening should be more prioritized to avoid diagnosis only in advanced stages. Moreover, the consumption of tobacco products needs to be addressed in parallel with risk factors, for example by raising tobacco taxes.

## Supplementary Information

Below is the link to the electronic supplementary material.

Supplementary Material 1

Supplementary Material 2

Supplementary Material 3

Supplementary Material 4

## Data Availability

Data sharing is not applicable to this article as no datasets were generated or analyzed during the current study.

## Abbreviations

Bs: Venezuelan Bolívares
CHEERS: Consolidated Health Economic Evaluation Reporting Standards statement
CT: Computer Tomography
DALY: Disability-Adjusted Life Year
GDP: Gross Domestic Product
GDPD: Gross Domestic Product deflator
GLOBOCAN: Global Cancer Observatory
GNI: Gross National Income
HCA: Human Capital Approach
HNL: Honduran Lempira
IHME: Institute for Health Metrics and Evaluation
ISPOR: International Society for Pharmacoeconomics and Outcomes Research
LIC: Low-income country
LMIC: Lower-middle income country
N: Number
OOP: Out-of-the pocket expenditure
PAR: Population-attributable risk
P-G: Therapy with cisplatine + gemcitabine
P-V: Therapy with cisplatin + vinerolebine
PET-CT: Positron emission tomography-computed tomography
PPP: Purchasing Power Parity
PRISMA: Preferred Reporting Items for Systematic Reviews and Meta-Analyses
PubMed: Public Medicine
PVFLP: Present Value of Future Lost Productivity
SAF: Smoking-attributable fraction
TND: Tunisian Dinar
USD: United States dollar
VN$: Vietnamese Dollars
WHO: World Health Organization
VSL: Value of a Statistical Life
VSLY: Value of a Statistical Life Year
YLL: Years of Life Lost
YPLL: Years of Productive Life Lost
