# Supplementary material for: The economic burden of lung cancer in low- and lower-middle-income countries: a systematic review
Source: Arch Public Health. 2025 Oct 13;83:243. doi: 10.1186/s13690-025-01738-6 (PMC12516874; doi:10.1186/s13690-025-01738-6)
Supplement: Supplementary file 1 — Supplementary Material 1 [file 13690_2025_1738_MOESM1_ESM.docx]

**Supplementary Table 1**: Search Terms divided into three categories and combined with “AND”

| **Economic Burden** | (Costs and Cost Analysis[MeSH] OR Health Care Costs[MeSH] OR Cost of Illness[MeSH] OR Hospital Costs[MeSH] OR Sick Leave[MeSH] OR Health Status Indicators[MeSH] OR Economics, Medical[MeSH] OR Health Expenditures[MeSH] OR Psychology, Industrial[MeSH] OR Socioeconomic Factors[MeSH] OR cost*[Title/Abstract] OR cost analysis*[Title/Abstract] OR cost comparison*[Title/Abstract] OR cost-minimization analysis*[Title/Abstract] OR pricing[Title/Abstract] OR affordability[Title/Abstract] OR health care cost*[Title/Abstract] OR healthcare cost*[Title/Abstract] OR health cost*[Title/Abstract] OR medical care cost*[Title/Abstract] OR treatment cost*[Title/Abstract] OR cost of illness[Title/Abstract] OR illness cost*[Title/Abstract] OR sickness cost*[Title/Abstract] OR illness burden*[Title/Abstract] OR disease cost*[Title/Abstract] OR hospital cost*[Title/Abstract] OR disability leave*[Title/Abstract] OR sick day*[Title/Abstract] OR illness day*[Title/Abstract] OR health status indicator*[Title/Abstract] OR health status index*[Title/Abstract] OR health expenditure*[Title/Abstract] OR economic burden[Title/Abstract] OR economic[Title/Abstract] OR medical economics[Title/Abstract] OR fee[Title/Abstract] OR funding*[Title/Abstract] OR finance*[Title/Abstract] OR financial management[Title/Abstract] OR absenteeism[Title/Abstract] OR presenteeism[Title/Abstract] OR efficiency[Title/Abstract] OR employment[Title/Abstract] OR unemployment[Title/Abstract] OR work day*[Title/Abstract] OR labor market[Title/Abstract] OR labor supply[Title/Abstract] OR indirect cost*[Title/Abstract] OR indirect expenditure*[Title/Abstract] OR direct cost*[Title/Abstract] OR direct expenditure*[Title/Abstract] OR out-of-pocket cost*[Title/Abstract] OR out-of-pocket expenditure*[Title/Abstract] OR cost burden[Title/Abstract] OR medication cost*[Title/Abstract] OR therapy cost*[Title/Abstract]) |
| --- | --- |
| **Lung Cancer** | ("Lung Neoplasms"[MeSH] OR "Bronchial Neoplasms"[MeSH] OR Carcinoma, Non-Small-Cell Lung[MeSH] OR Small Cell Lung Carcinoma[MeSH] lung neoplasm*[Title/Abstract] OR pulmonary neoplasm*[Title/Abstract] OR lung cancer[Title/Abstract] OR lung carcinoma*[Title/Abstract] OR pulmonary carcinoma*[Title/Abstract] OR bronchial neoplasm*[Title/Abstract] OR bronchiogenic carcinoma*[Title/Abstract] OR respiratory tract neoplasm*[Title/Abstract] OR pulmonary cancer*[Title/Abstract] OR cancer of the lung[Title/Abstract] OR cancer of lung[Title/Abstract] OR SCLC[Title/Abstract] OR small cell lung cancer[Title/Abstract] OR small cell lung carcinoma*[Title/Abstract] OR oat cell lung cancer[Title/Abstract] OR oat cell carcinoma*[Title/Abstract] OR NSCLC[Title/Abstract] OR non small cell lung carcinoma*[Title/Abstract] OR nonsmall cell lung carcinoma*[Title/Abstract] OR non-small cell lung carcinoma*[Title/Abstract] OR squamous cell carcinoma*[Title/Abstract] OR large cell carcinoma*[Title/Abstract] OR lung adenocarcinoma*[Title/Abstract] OR alveolar adenocarcinoma*[Title/Abstract] OR alveolar carcinoma*[Title/Abstract] OR alveolar cell carcinoma[Title/Abstract]) |
| **Low-and Low-Middle-income Countries** | ("Developing Countries"[MeSH] OR "Africa South of the Sahara"[MeSH] OR "Asia, Southeastern"[MeSH] OR "Asia, Central"[MeSH] OR "Asia, Western"[MeSH] OR "South America"[MeSH] OR developing countr*[Title/Abstract] OR least developed countr*[Title/Abstract] OR less developed countr*[Title/Abstract] OR under-developed nation*[Title/Abstract] OR under developed nation*[Title/Abstract] OR third-world countr*[Title/Abstract] OR developing nation*[Title/Abstract] OR low-income countr*[Title/Abstract] OR lower-middle-income countr*[Title/Abstract] OR low and middle income countr*[Title/Abstract] OR low-middle income countr*[Title/Abstract] OR high-middle income countr*[Title/Abstract] OR resource-limited setting*[Title/Abstract] OR low resource[Title/Abstract] OR low-resource[Title/Abstract] OR LMIC[Title/Abstract] OR LLMIC[Title/Abstract] OR Afghanistan[Title/Abstract]) OR (Angola[Title/Abstract]) OR (Bangladesh[Title/Abstract]) OR (Benin[Title/Abstract]) OR (Bhutan[Title/Abstract]) OR (Bolivia[Title/Abstract]) OR (Burkina Faso[Title/Abstract]) OR (Burundi[Title/Abstract]) OR (Cambodia[Title/Abstract]) OR (Cameroon[Title/Abstract]) OR (Central African Republic[Title/Abstract]) OR (Chad[Title/Abstract]) OR (Comoros[Title/Abstract]) OR (Congo[Title/Abstract]) OR (Côte d’Ivoire[Title/Abstract]) OR (Djibouti[Title/Abstract]) OR (Egypt[Title/Abstract]) OR (Eritrea[Title/Abstract]) OR (Eswatini[Title/Abstract]) OR (Gambia[Title/Abstract]) OR (Ghana[Title/Abstract]) OR (Guinea[Title/Abstract]) OR (Guinea-Bissau[Title/Abstract]) OR (Haiti[Title/Abstract]) OR (Honduras[Title/Abstract]) OR (India[Title/Abstract]) OR (Jordan[Title/Abstract]) OR (Kenya[Title/Abstract]) OR (Kiribati[Title/Abstract]) OR (Korea[Title/Abstract]) OR (Kyrgyz Republic[Title/Abstract]) OR (Lao PDR[Title/Abstract]) OR (Lebanon[Title/Abstract]) OR (Lesotho[Title/Abstract]) OR (Liberia[Title/Abstract]) OR (Madagascar[Title/Abstract]) OR (Malawi[Title/Abstract]) OR (Mali[Title/Abstract]) OR (Mauritania[Title/Abstract]) OR (Micronesia[Title/Abstract]) OR (Morocco[Title/Abstract]) OR (Mozambique[Title/Abstract]) OR (Myanmar[Title/Abstract]) OR (Namibia[Title/Abstract]) OR (Nepal[Title/Abstract]) OR (Nicaragua[Title/Abstract]) OR (Niger[Title/Abstract]) OR (Nigeria[Title/Abstract]) OR (Pakistan[Title/Abstract]) OR (Papua New Guinea[Title/Abstract]) OR (Philippines[Title/Abstract]) OR (Rwanda[Title/Abstract]) OR (São Tomé and Príncipe[Title/Abstract]) OR (Senegal[Title/Abstract]) OR (Sierra Leone[Title/Abstract]) OR (Solomon Islands[Title/Abstract]) OR (Somalia[Title/Abstract]) OR (South Sudan[Title/Abstract]) OR (Sri Lanka[Title/Abstract]) OR (Sudan[Title/Abstract]) OR (Syrian Arab Republic[Title/Abstract]) OR (Tajikistan[Title/Abstract]) OR (Tanzania[Title/Abstract]) OR (Timor-Leste[Title/Abstract]) OR (Togo[Title/Abstract]) OR (Tunisia[Title/Abstract]) OR (Uganda[Title/Abstract]) OR (Uzbekistan[Title/Abstract]) OR (Vanuatu[Title/Abstract]) OR (Vietnam[Title/Abstract]) OR (West Bank and Gaza[Title/Abstract]) OR (Yemen[Title/Abstract]) OR (Zambia[Title/Abstract]) OR (Zimbabwe[Title/Abstract]) |
