# Supplementary material for: The economic burden of lung cancer in low- and lower-middle-income countries: a systematic review
Source: Arch Public Health. 2025 Oct 13;83:243. doi: 10.1186/s13690-025-01738-6 (PMC12516874; doi:10.1186/s13690-025-01738-6)
Supplement: Supplementary file 3 — Supplementary Material 3 [file 13690_2025_1738_MOESM3_ESM.docx]

**Supplementary Table 3**: Description of type of costs

| **Authors [Country, Reference]** | **Type of costs** |
| --- | --- |
| **Khatiwoda S R, et al. [Nepal, 39]** | Medical costs comprised of costs of consultation, diagnosis, investigations, hospital care and treatment taken such as chemotherapy, radiotherapy, surgery, or palliative and supportive care  Non-medical cost comprised of costs of food, travel, and accommodation during health service utilization  Direct costs calculated as medical and non-medical costs |
| **Hoang Anh PT, et. al [Vietnam, 40]** | Direct costs combined as medical and non-medical costs  Medical costs such as service fees, overhead costs, drugs not included in the service fees  Non-medical costs included transportation and supplemental foods  Indirect costs comprised patients’ income loss due to sick leave and premature death, as well as income losses for family members providing patient care |
| **Ross H, et al. [Vietnam, 41]** | Direct costs presented primarily in the form of higher healthcare costs  Indirect costs related to productivity losses as a result of morbidity and premature mortality |
| **Pearce A, et al. [India, 42]** | Indirect costs calculating lost productivity and calculates the present value of potential time in the workforce (the measure of productivity) using market wages. It calculates losses only for the period it takes to replace a worker in the workplace, known as the friction period |
| **Fenniche S, et al. [Tunisia, 43]** | Costs were calculated for hospitalization, endoscopic, anatomopathological and imaging examinations, antimitosis treatment and radiotherapy |
| **Tachfouti N, et al. [Morocco, 44]** | Costs for diagnosis including physical examination, Chest X-ray, liver function tests and liver ultrasound and bone scan  Costs for treatment depending on cancer stage, such as Surgical resection, chemotherapy, radiotherapy, and a follow-up  Costs for postoperative treatment such as platinum, radiotherapy, and follow-up |
| **Pichon-Riviere A, et al. [Bolivia and Honduras, 45]** | Direct medical costs including diagnosis, treatment, and follow-up |
| **Harizi C, et al. [Tunisia, 46]** | Cost components are hospitalization, biological examinations, imaging, chemotherapy, surgery, and radiotherapy |
| **Kyaing N, et al. [Myanmar, 47]** | Cost per visit, cost per admission and total costs |
| **Faruque G M, et al. [Bangladesh, 48]** | Total costs, average cost of treatment, cost per outpatient visit, cost per admission |
| **Wahab KA, et al. [Egypt, 49]** | Indirect costs were estimated as productivity losses from premature mortality due to five cancer types (liver, lung, breast, bladder, and cervical cancer).  The Human Capital Approach was used to calculate Years of Life Lost (YLL), Years of Productive Life Lost (YPLL), and the Present Value of Future Lost Productivity (PVFLP), using national life expectancy, retirement age, labor force participation, and income data. |
| **Rashdan O. [Jordan, 50]** | Indirect costs were calculated as the monetary value of productivity losses due to premature mortality and morbidity for 22 cancer types, using Disability-Adjusted Life Years (DALYs) from the Global Burden of Disease Study.  Two valuation methods were applied: the Human Capital Approach (valuing each DALY at gross national income per capita) and the Value of a Statistical Life Year approach (adjusted for purchasing power parity). |
| **El Harch, et al [Morocco, 51]** | Direct medical costs included hospitalization, diagnostic imaging, laboratory tests, specialist consultations, surgery, chemotherapy, radiotherapy, targeted therapy, immunotherapy, and medications. |
| **Olumide AO, et al. [Nigeria, 52]** | Direct medical costs included costs of hospital admission, medications, diagnostics, and clinic visits for terminal in-patient care. Direct non-medical costs included transportation expenses. |
| **Mwai D, et al. [Kenya, 53]** | Direct costs included medical expenses along the continuum of care for tobacco-related illnesses, such as medication, diagnostic tests, hospital services, and outpatient care, estimated using an Activity-Based Costing approach.  Indirect costs comprised productivity losses due to morbidity and premature mortality, calculated using patient-reported income loss and the Tobacco Attributable Factor to derive the proportion of costs attributable to tobacco use. |
